# Evaluation of Long-Term Flow Controller for Monitoring Gases and Vapors in Buildings Impacted by Vapor Intrusion

**DOI:** 10.3390/ijerph20064811

**Published:** 2023-03-09

**Authors:** Alan Rossner, David P. Wick, Christopher Lutes, Benjamin Stone, Michelle Crimi

**Affiliations:** 1 Institute for Sustainable Environment, Clarkson University, Potsdam, NY 13699, USA; 2 School of Individualized Study, Rochester Institute of Technology, Rochester, NY 14623, USA; 3 Jacobs Engineering Group Inc., Dallas, TX 75201, USA

**Keywords:** time-integrated sampling, volatile organic compounds, capillary flow controller, diaphragm flow controller, Summa canister

## Abstract

This study evaluated the use of a long-term capillary flow controller paired with an evacuated canister for indoor air exposure monitoring in a vapor intrusion (VI) environment with trichloroethylene in comparison to the traditional method utilizing a diaphragm flow controller. Traditionally, air sampling with 6 L evacuated canisters equipped with diaphragm flow controllers has been best suited for 8 to 24 h samples. New advances in capillary flow controllers can extend sampling to up to 3 weeks by reducing flow rates to 0.1 milliliters min^−1^. During six 2 wk sampling events, conventional diaphragm flow controller canisters were used to collect 24 h samples simultaneously with capillary flow controllers collecting 2 wk samples. Testing was performed at four indoor locations in buildings impacted by VI with co-located samples for each method at each location. All samples were analyzed using GC/MS, and the results were statistically analyzed to produce a direct comparison of the two sampling systems. Ninety-two percent of the 14 d capillary samples were within the 95% levels of agreement of the average concentration of the diaphragm flow controllers. The ability to collect 14 days of data, with less occupant disturbance, allows for improved exposure assessments and thus improved risk management decisions.

## 1. Introduction

The potential impact of indoor air quality on human health is important to examine because of the amount of time individuals spend inside, and typically indoor air pollutants occur in higher concentrations than outdoor pollutants. Sources of pollution may exist within or outside the building and can accumulate above or below the ground. Whatever the source of pollution, indoor air quality testing is necessary to characterize exposures and risks to building occupants [1]. In buildings impacted by vapor intrusion (VI), indoor air quality can result in an occupant’s increased risk for developing adverse health effects from long-term exposure to low-part-per-billion (ppb) or lower concentrations of volatile organic compounds (VOCs) in indoor air [2,3,4,5,6]. Assessing long-term exposure from VI is challenging, as temporal and spatial variability can cause day-to-day VOC concentrations to shift by orders of magnitude [7,8]. Furthermore, background VOCs emitted by human activities, building materials, solvents, and contaminated well water can make identifying VI exposure sources difficult [9,10,11]. 

Current US EPA guidance on VI assessments calls for a multiple-lines-of-evidence approach for predicting VI risk through the collection of groundwater, soil gas, and indoor air samples [12,13,14,15]. Because indoor air samples provide the clearest insight into an occupant’s exposure, they are generally weighted most heavily in making risk-based decisions, even though they incorporate the effects of both vapor intrusion and indoor sources. While guidance for indoor air sampling can vary by geographical location, most sampling approaches call for the collection of one to four 24 h indoor air samples over a 2 wk time period in different seasons [16,17]. Developing a sampling strategy that is representative of an occupant’s long-term exposure is limited by the uncertainty surrounding the dominant driving forces for VI; thus, a long-term time-weighted average can provide a more reliable characterization of VI exposure for chronic effects [18].

Rappaport discusses the value of a long-term average as the most effective means for predicting long-term disease, and that short intermittent exposures to high concentrations (peak values) are, in general, less relevant to chronic disease rates or patterns [19]. However, if the peak exposure is significantly larger (~2 orders of magnitude) than the median concentration, the long-term time-weighted average can be heavily influenced by a small percentage of peak time periods. Holton [3] and Schuver [20] presented findings that 25 of 723 days sampled (~3.5 % of the sampling days) made up 50% of the long-term average in one indoor air case influenced by a preferential pathway. While Holton and Schuver’s work focused on the health impact of peak values and Rappaport’s work draws a general conclusion that the long-term average is the driver of disease, the key issue for both is the magnitude and duration of the “peak values.” Long-term sampling (14 d) provides more information, yet high transient concentrations (peak values) can still affect the overall time-weighted average. Hence, longer-term air sampling will increase the odds of observing the upper level of the exposure distribution for a specific location or for a group of building occupants with an economically viable number of samples. 

Obtaining a long-term average requires the collection of multiple air samples, which can be costly and time-consuming and can delay the decision process. There are two conventional air-sampling methods currently being widely used in VI investigations: passive diffusion tubes (either thermally desorbed (TD) or solvent-extracted) and evacuated air-sampling canisters equipped with flow controllers. While active sampling systems with sorbents can be used, they are logistically challenging for long periods of time, as they require greater attention. An important consideration is that TD tubes and canister-flow controller systems allow for the passive collection of air samples for extended periods of time without the use of air-sampling pumps [21,22,23,24,25]. 

Canisters, regardless of the flow controller employed, are useful for the sampling of many compounds across a wide range of functional groups with approved sampling and analytical methods, i.e., TO-15 and NIOSH 3900, resulting in a good recovery for C1 to C10 [26,27,28,29,30]. There is limited impact by the environmental conditions of temperature and humidity on the canister, although water condensation can affect the recovery of polar compounds. Sample stability ranges from weeks to months. Although somewhat bulky to transport, canisters are easy to thoroughly clean and can last for more than 10 years. Canisters allow for easy-to-perform dilutions and re-analyses, providing substantial flexibility in the concentration range and compound analysis. Reactions inside the canisters do occur for select compounds, such as limonene, ozone, and formaldehyde, and therefore are not appropriate for canister sampling. 

LeBouf et al. studied the use of canisters for the collection of VOCs [31]. Their study demonstrated that canisters could effectively collect C1-C10 of VOCs at concentrations from sub-ppb to ppm levels while keeping samples stable enough for acceptable recoveries following the US EPA TO-15 protocol for the analysis of VOCs. The coating on state-of-the-art canisters prevents many VOCs from reacting with canister walls and allows for high stability for up to a month or longer. Canisters provide detection limits in the part per trillion range and often display precisions of less than 15% for re-analysis, which is within the ASTM criteria of +/− 30% [32,33,34,35]. The acceptance range in commonly conducted inter-laboratory comparison studies of method TO-15 using replicate samples has demonstrated considerably wider ranges [32].

**Figure 1:**
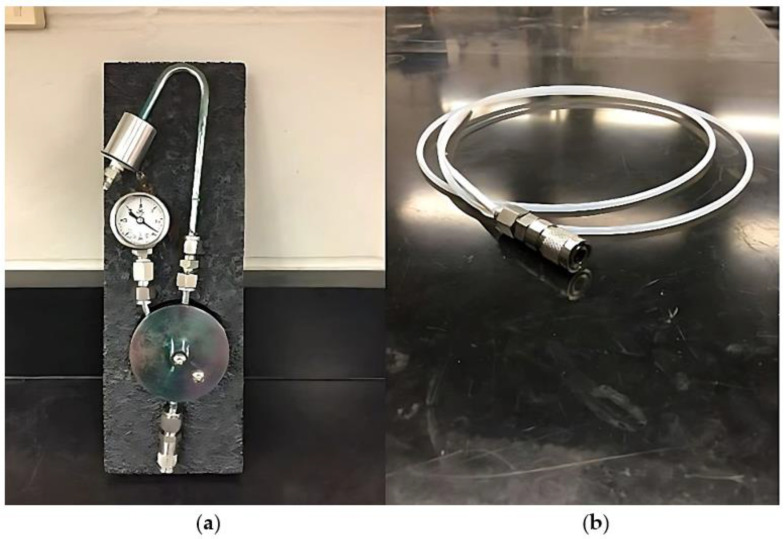
(**a**) A typical diaphragm flow controller can regulate airflow rates as low as 2.8 mL/min to 3.5 mL/min but can be adjusted to achieve much higher flow rates. (**b**) A typical capillary flow controller, with a small inside diameter of 0.05 mm, functions as a restricting orifice to maintain a low sample flow rate (0.1 to 0.5 mL/min), permitting an extended sampling time. The specific capillary flow controllers used in this research had mean flow rates that were typically 0.11 mL/min and 0.31 mL/min.

Integrated sampling with evacuated canisters requires the use of a flow controller to regulate the volume of air entering the canister over time. Current flow controllers (diaphragms, as shown in Figure 1a) restrict airflow in a range from 3 to 4 milliliters per minute (mL/min), hence allowing for one 24 h sample with a 6 L canister. The diaphragm flow controller maintains a relatively constant flow rate using a critical orifice that acts as a flow restrictor upstream of a diaphragm controlled by a mechanical spring. The pressure differential across the diaphragm is the result of outside atmospheric pressure and internal canister pressure [36]. The diaphragm weighs about 0.45 kg and varies in length from 15 to 20 cm long. Current designs do not allow for flow rates below 3.0 mL/min, which may be a function of the current spring design. Attempts to use diaphragm controllers to achieve greater run times (24–48 h) have been made but frequently result in less reliable flow control/duration results [37]. 

Multiple 24 h samples are needed for a longer-term integrated average (weeks), and as a result, this strategy can be costly and require additional building occupant disturbance due to repeated site visits. As a means to extend the sampling time and reduce sampling costs, a long-term capillary flow controller (Figure 1b) was developed in the late 1990s to further reduce the airflow rate into an evacuated canister [38,39]. The capillary flow control device is a passive controller that allows contaminated air to pass into the canister at low flow rates (0.1 to 0.5 mL/min). A fused silica capillary column (inside diameter of 0.05 mm) functions as a restricting orifice to maintain a low flow rate of air. The resulting flow rate is a function of the capillary diameter and length [38]. The capillary flow controller has been tested under controlled laboratory conditions at varying concentrations of numerous VOCs, as well as a variety of temperatures and humidity [35]. In addition, the flow controllers have been tested as personal sampling devices (with smaller canisters) and area sampling devices in industrial environments [36]. 

In 2016, a capillary flow controller was first commercialized by Restek, Inc. (Bellefonte, PA, USA) [36]. The flow controller consists of a 15 cm long capillary housed inside a 0.32 cm diameter Teflon tube weighing less than 30 g. All previous capillary flow controllers were lab prototypes and evaluated over several years in lab and field studies [38,39,40]. The evaluation of the performance of the first commercialized version is the focus of this study. 

The objective of this field investigation was to evaluate and compare the performance of the long-term capillary-canister system used to collect a 2-week-long air sample to 14 consecutive 24 h samples collected by the traditional diaphragm canister method. Testing was conducted at a range of temperatures for trichloroethylene concentrations in unoccupied and unheated buildings impacted by VI. 

A less-intrusive 14-day sampling method, as used in this study, should allow for improved exposure assessments and thus improved risk management decisions. The replication of this methodology will allow for long-term sample collection in a variety of industrial and community environments and across a full range of analytes and concentrations of interest in indoor environments. For the remainder of this article, the capillary flow controller equipped with a canister will be referred to as a capillary flow controller, while comparative diaphragm canister sampling systems will be referred to as diaphragm flow controllers.

## 2. Materials and Methods

### 2.1. Sampling Environment 

**Figure 2:**
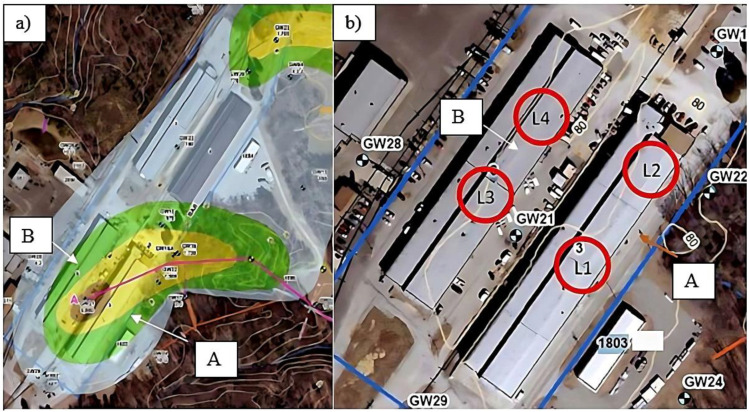
(**a**) Buildings (A and B) chosen for sampling depicting the contamination of groundwater beneath each building (brown = most contamination; yellow = medium contamination; green = low contamination; and gray = possible contamination). (**b**) An enlarged view of the buildings with approximate sampling locations labeled L1–L4 and circled in red. Note: GW labels represent some of the groundwater monitoring wells installed several years prior to this study.

Seasonal ambient and indoor air samples were collected in a location within the mid-south Atlantic region in buildings with a known history of VI. Past screening of the site identified chlorinated VOCs as primary compounds in both groundwater and indoor air. Indoor air samples were collected in two large unoccupied buildings (Buildings A and B), each approximately 50,000 square feet in area and constructed circa 1919 and 1941, respectively. Both buildings were constructed on concrete slabs, with Building B on an elevated slab. Each building included a mix of warehouse-style open shops and subdivided smaller rooms, including offices and break rooms. There were no building-wide climate control ventilation systems within the two buildings when they were occupied, nor were any forced air ventilation or exhaust systems operating at the time of this study. The buildings were not insulated, and several areas within the buildings were exposed to outside air via cracks and openings in the walls and roofs. The buildings consisted of concrete slab floors, cement block walls, metal sheeting, and wood roofs. Several locations contained floor drains and cracks in both Buildings A and B. Electrical conduits could have provided preferential pathways for VI into the buildings. Vapor intrusion in Building B was primarily attributed to interior block walls and a small utility chase with a dirt floor and conduits leading down into the dirt serving as preferential pathways. At the time of sampling, the buildings had been unoccupied, and no indoor air contaminant sources were found in the buildings. Both buildings are located directly above an identified contaminated groundwater plume (trichloroethylene (TCE)), with the majority of contamination beneath Building A (see Figure 2). Sections of Building B are outside the groundwater plume boundaries. Ambient air samples were collected outside one of the buildings on a loading dock located away from traffic and upwind of the buildings. 

Sampling was conducted in six separate events from March 2017 to August 2018, with each event lasting 2 weeks. For each sampling event, the same four indoor locations and one ambient (outside) location were monitored for 14 days. Indoor locations were labeled as Building A-L1 and -L2 and Building B-L3 and -L4. In each location, SilcoCan (Restek Corp., Bellefonte, PA, USA) and Silonite (Entech Instruments, Simi Valley, CA, USA) air canisters were placed side-by-side approximately 1.0 m off the floor and within a 0.5-m radius of each other. Additional details with respect to the sample collection plan are available in Supplemental Materials.

### 2.2. Temporal and Spatial Conditions 

Diaphragm flow controllers were used to collect 24 h samples, while capillary flow controllers were simultaneously used to collect 14 d samples. In total, fourteen 24 h samples and three 14 d samples were collected during each 2 wk period at each location. In addition, to assess the field precision of the diaphragm samplers, an additional 24 h co-located sampler was sequentially rotated through the 4 locations on a daily basis for the duration of the 14 d sampling events. Additionally, 24 h sub-slab samples were collected from each location into 1 L canisters using a capillary flow controller with a flow rate of 0.3 mL/min. The four canister devices in each location included one 6 L canister equipped with a diaphragm flow controller (Entech Instruments, Simi Valley, CA, USA) that regulated airflow between 2.8 and 3.5 milliliters per minute (mL/min), one 6 L canister equipped with a 0.11 mL/min capillary flow controller (Restek Corp.), and two 15 L canisters equipped with 0.31 mL capillary flow controllers (Restek Corp.). Both the capillary flow and diaphragm flow controllers were connected to the canisters using a Swagelok quick release (Swagelok, Solon, OH, USA), as shown in Figure 1.

When this research sampling plan was designed, the commercially available capillary flow controllers did not have a low enough flow rate to sample for a full two weeks in a 6 L canister; hence, 15 L canisters were chosen. However, as the first air-sampling event started, Restek was able to deliver capillary flow controllers that allowed for 2 weeks of sampling in 6 L canisters. The sampling plan was modified to include both 15 L canisters and 6 L canisters with the capillary flow controllers. Diaphragm flow controllers were used on 6 L canisters as the standard sampling method for use in buildings with vapor intrusion concerns.

Environmental conditions were monitored indoors using temperature (°C) and relative humidity (%) data-logging probes (Lascar Electronics, Erie, PA), and outdoor conditions were obtained from Weather Underground for station KPHF (Newport News VA). 

Evacuated air canisters were prepared prior to sampling by thorough batch cleaning, which involved flushing and pressurizing with ultra-high-purity (UHP)-grade nitrogen (N_2_) through a process of multiple cycles. All canisters were then analyzed to ensure they were clean, and if not, additional cleaning cycles were completed. After ensuring no residual compounds remained, canisters were then evacuated to below 50 mTorr before sampling. In the field, internal canister pressures were measured and recorded using Ashcroft (Ashcroft, Stratford, CT, USA) model 2174 digital pressure gauges. Pressure gauges were located on all of the canisters with the capillary flow controllers to allow for tracking the pressure change over the 14 d sampling. The target pressure for the capillary-canister sampling system was 300 Torr, resulting in filling the canister to approximately 40 percent of the canister volume. The diaphragm controllers allow for a larger volume to be collected, with a target pressure of approximately 650 Torr equating to 85% of the canister volume. Canisters were not used if they had leaked during shipping. Upon completion of both the 24 h samples and 14 d samples, the canisters were pressurized using ultra-high-purity (UHP) Nitrogen before they were shipped to the laboratory for analysis to minimize the risk of contamination during shipping. The canister pressure was recorded prior to sampling, after sampling, and after pressurization with UHP-N2 to allow for the calculation of a dilution factor. 

All 24 h canisters collected in the field were shipped within 2 days of collection and analyzed within 1 week at the Clarkson University Center for Air Resources Engineering & Sciences (CARES) laboratory. To perform the validation of analytical accuracy, four to five canisters were chosen for inter-laboratory analysis for each of 5 rounds (March 2017 was not included) by Centek Laboratories (Syracuse, NY, USA), accredited by two national accreditation programs: National Environmental Laboratory Accreditation Program (NELAP) and American Industrial Hygiene Association Laboratory Accreditation Program (AIHA-LAP). 

The analysis was performed using a modified version of EPA TO-15 [26] for evacuated air canisters using a Markes International (Bridgend, United Kingdom) CIA Advantage preconcentrator connected in line with a Thermo Scientific Trace Gas Chromatograph (GC) Ultra (Serial No. 320060966)/DSQ mass spectrometer (MS) (Serial No. MS 110-0130). Compounds were desorbed onto a 30-m Rxi ^®^-5Sil MS column (30 m, 0.25 mm ID, 0.25 um film thickness) in sample volumes of 100 to 300 mL. Trap settings were set for a minimum temperature of 0 °C with a 20 °C per minute increase up to 300 °C, which was held for 5 min to remove carryover and was eventually followed by a 1 min purge of UHP N_2._ The flow path temperature was set to 120 °C, and the carrier pressure did not drop below 5 psi. The GC oven program was optimized for TCE and consisted of a 2 min hold at 40 °C, followed by a temperature ramp of 5 °C min ^−1^ to a final temperature of 90 °C for a total run time of 12 min. 

Sub-slab samples containing higher concentrations of VOCs (>1 ppm) were analyzed on a Hewlett Packard (Palo Alto, CA, USA) 5890 Series II GC flame ionization detector (FID) with a Zebron (Torrance, CA, USA) column (30 m × 0.53 mm I.D. and a 1.5 um film thickness, Serial No. 104399). No prefocusing was necessary due to the high concentrations in the sub-slab. The following conditions were used for the oven temperature program to maximize the peak area and run time: the initial temperature was set at 60 °C for 1 min followed by a 15 °C/min ramp to a final temperature of 135 °C. Hydrogen gas and breathing air Grade D were used as ignition sources for the FID. Ultra-high-purity Helium was used as the auxiliary gas. All compressed gases were obtained from AirGas (Brushton, NY, USA).

A calibration standard (cylinder) of common indoor air VOCs was used for this study, although once air sampling started, it became evident that TCE was the only compound of concern in the two buildings evaluated in this study. Five-point calibration curves were constructed for TCE (R^2^ was above 0.99), and the method detection limit for TCE was 0.03 ppb. Multiple calibration curves were used for the broad range of concentrations measured by GC/MS. Prior to every sampling campaign, new calibration curves were made to correct for instrument signal deviation. Quality control (QC) and quality assurance (QA) procedures were performed during analysis, including running blank samples and a 6 ppb calibration check standard prior to the first sample of each day to validate instrument performance. One sample canister out of eight was chosen at random for triplicate analysis per day of analysis. 

Data were managed using Microsoft Excel, and descriptive statistics were calculated using IBM SPSS Statistics 25 (IBM Corp., Armonk, NY, USA). Jmp Statistics 9 JMP (13.2) Software (SAS Institute Inc., Cary, NC, USA) was used to complete multivariate analysis. A test for normality was conducted based on the Shapiro–Wilk test. Comparative analysis tests were conducted on the pairwise data based on the sample period and location through a series of methods, including linear regression and analysis of variance (ANOVA). The 24 h samples collected in each location were used to generate a 14 d compound-specific average concentration. Success criteria are met if the 14 d sample falls within the 95% confidence interval of the mean of the 24 h samples for each location. The 95% confidence interval around the mean of the set of 24 h samples describe the likelihood that the mean of a separately collected set of fourteen samples (same sample location, same dates, and same laboratory) would have a mean similar to the mean calculated for the original set of fourteen samples. Thus, comparing the result of the capillary controller sample to that confidence interval, or what could be referred to as 95% upper and lower levels of agreement, is an estimate of whether the two types of controllers produce equivalent estimates of airborne concentrations over a two-week period. The diaphragm flow controller was used as a reference or guide to the “actual” mean and confidence intervals of all TCE samples collected at each sample location. To assess whether the data were normal or lognormal, a Shapiro–Wilk test was run on the 24 h data from each location and each sampling event (24 sets of data). The results of the test for normality allowed for the use of lognormal confidence intervals when comparing the two flow controllers. 

## 3. Results

Sampling occurred for two weeks each in March 2017, May 2017, August 2017, January 2018, May 2018, and August 2018; the timing was chosen to align with the seasons at the site. Outdoor weather conditions during the sampling periods ranged from −16.1 °C in January to 33.9 °C in August, while humidity levels were relatively high for all sampling events (>68%). Precipitation in the form of rain averaged around 2 inches per 14 d; however, there were heavy periods of rain with an accumulation of greater than 1 inch for two consecutive days in May 2017 and January 2018. Indoor and outdoor environmental conditions are shown in Table 1. Indoor temperatures and humidity levels were comparable to the outside temperature in all four locations. 

Including site samples, blanks, and quality controls, over 800 canisters were analyzed. Analyses of indoor diaphragm samples show that both buildings are heavily impacted by TCE in indoor air. Ambient (outdoor) samples resulted in no significant detection of any other VOCs, indicating that contamination is not coming from outside the buildings and that the TCE concentrations were a result of vapor intrusion. TCE concentrations were greater than 0.1 ppb, while the ambient samples were less than the minimum detection limits (MDLs). During the study period, the individual 24 h measurements of TCE ranged from 0.1 to 144 ppb and the 14 d averages ranged from 1.31 to 49.5 ppb for all locations. The focus of this research was to use the TCE concentrations to compare the performance of the capillary to the diaphragm flow controllers. 

Table 2 shows the mean indoor concentrations of TCE for each location for all six of the two-week sampling events. The Wilk–Shapiro test for normality indicated a combination of normal and lognormal distributions for the datasets; hence, both arithmetic and geometric means are included. The data demonstrate the wide range of indoor concentrations (>10×) for all of the 24 h samples by location. As outlined by the 2007 ITRC report “Vapor Intrusion Pathway: A Practical Guideline” [41], spatial correlation analysis is a tool used to distinguish between background and VI exposure pathways. Clear spatial variability within each of the four indoor locations was consistent with the underlying groundwater plume, as the mean indoor concentration of TCE was the highest in Bldg A-L1 and the lowest in Bldg B-L4. From previous monitoring at the site, L1 is located directly above the highest contaminated area, whereas L4 is outside the groundwater plume. L4 experiences enhanced air exchange, as some parts of the roof have been removed. In general, indoor TCE concentrations were 6 to 10 times higher at Bldg A-L1 vs. Bldg B-L4. Over the six sampling events, samples were collected on 84 distinct days. Analysis showed arithmetic and geometric mean 24 h TCE concentrations ranging from a low of 1.3 to 1.7 ppb in January 2018 at L4 to a high of 38.1 to 49.5 ppb in May of 2017 at L1. 

**Figure 3:**
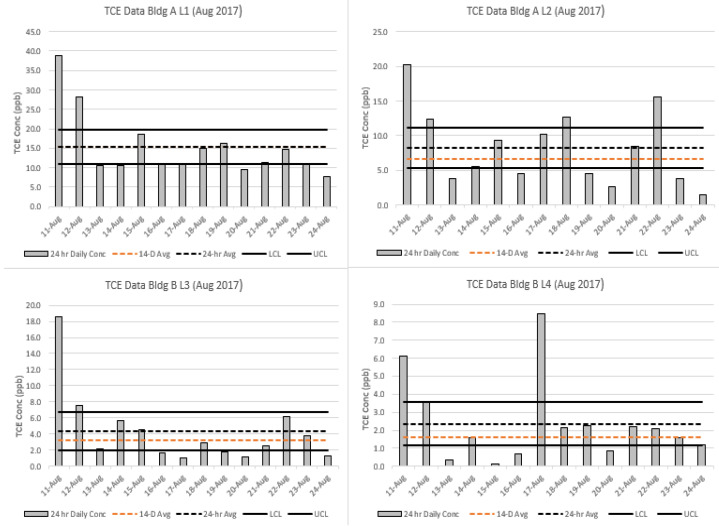
Comparative analysis of TCE concentrations from four locations (L1, L2, L3, L4) during a 2 wk period in August 2017 using traditional diaphragm (24 h) and capillary (14 d) controllers. Vertical gray bars represent daily concentrations, gray dashed lines represent the geometric mean (GM) of the daily (24 h) concentrations, solid black lines represent the 95% upper and lower confidence levels for the GM of the daily concentrations, and the orange dashed line represents the 14 d GM. Note: In the L1 plot, the two dashed lines are superimposed (the GMs are almost identical).

**Figure 4:**
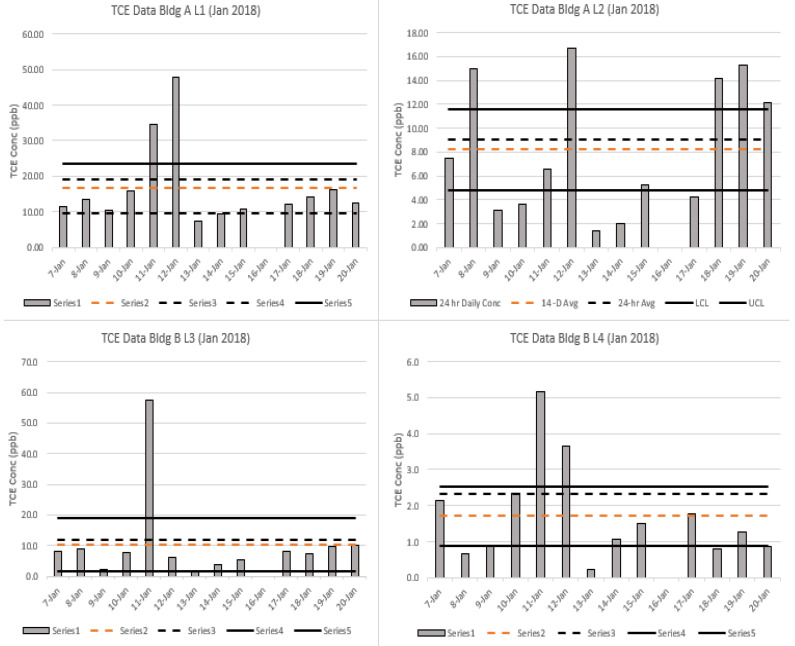
Comparative analysis of TCE concentrations from four locations (L1, L2, L3, L4) during a 2 wk period in January 2018 using traditional diaphragm (24 h) and capillary (14 d) controllers. Vertical gray bars represent daily concentrations, gray dashed lines represent the geometric mean (GM) of the daily (24 h) concentrations, solid black lines represent the 95% upper and lower confidence levels for the GM of the daily concentrations, and the orange dashed line represents the 14 d GM.

Figure 3 and Figure 4 show the daily 24 h TCE measurements using the traditional diaphragm flow controller, along with the 14 d measurements using the capillary flow controller for August 2017 and January 2018, respectively. The vertical bars show the 24 h daily concentrations collected with the diaphragm controller. The orange dotted line shows the 14 d sample concentration, and the gray dotted line shows the average of the 14 daily samples (24 h). As discussed in the methods, success criteria are met if the 14 d sample falls within the 95% upper and lower levels of agreement of the mean of the 24 h samples for each location (solid black lines). As shown in Figure 3 and Figure 4 (and Supplemental Figures S1–S4), the 14 d averages fall within the 95% CI for the 24 h samples for 22 of the 24 events, demonstrating good agreement between the two methods. 

It is important to note in Figure 3 and Figure 4 (and Supplemental Figures S1–S4) that many (55%) of the fourteen 24 h samples lie outside the confidence interval around the mean. For example, in Figure 3, Building A-L1 shows that 8 of the 14 individual 24 h samples lie outside of the confidence interval around the mean. Thus, in actual practice, when financial and sampling intrusiveness considerations often result in only one or two 24 h samples being taken during any given season, the mean exposure will typically be poorly estimated by the current (diaphragm) sampling approach. 

**Figure 5:**
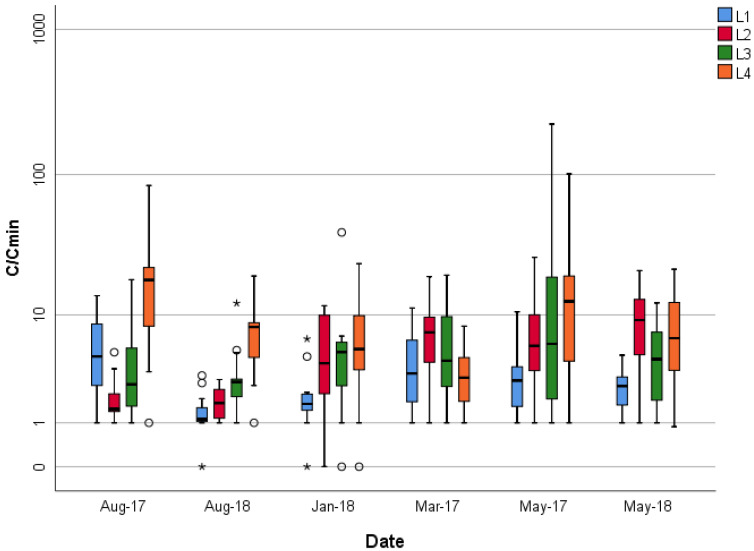
A series of box plots demonstrating the ratio of the minimum concentration to each corresponding concentration for the respective 14 d sampling event. The ratio was determined for each of the 24 sampling events. The x-axis represents the dates and locations of the sampling events; the y-axis is the ratio of the lowest concentration to each respective concentration for a specific date and location. Note: the center lines are the medians, the bottom and top of the boxes are the 25th and 75th percentiles, the whiskers are the 95th percentile, circles are between 1.5 and 3.0 of the interquartile (IQR) lines, and the asterisks are extreme values greater than 3 IQRs. Not all datasets have values above 1.5 IQR.

Temporal variability was observed within and between the six sampling events, as shown in the box-and-whisker plot in Figure 5, comparing the observed concentration as a ratio of the minimum concentration to all other concentrations (C/C_min_) for each sampling event at each location for the 24 h samples. This dimensionless ratio created a dataset comparing the lowest concentration to each concentration collected during the 14 d sampling event. This analysis was conducted to examine the variability with respect to the minimum concentration for each sampling period. Presenting the data in this form allows one to observe the variability in TCE concentrations with each 14 d period and between the six different sampling events. The 24 sets of 14 single-day measurements showed an order of magnitude or more shift in concentration in 20% of the samples (5 box plots exceeding 10 times the lowest concentration for the 14 d dataset), and 1% displayed two orders of magnitude shifts within the two-week period (Figure 5), thus demonstrating that the capillary flow controller performed well when airborne concentrations fluctuated by >10 times during a sampling period. These fluctuations in airborne concentrations are consistent with previous studies evaluating VI concentrations and environmental conditions, such as the temperature differential and heavy rainfall [3,41,42]. 

**Figure 6:**
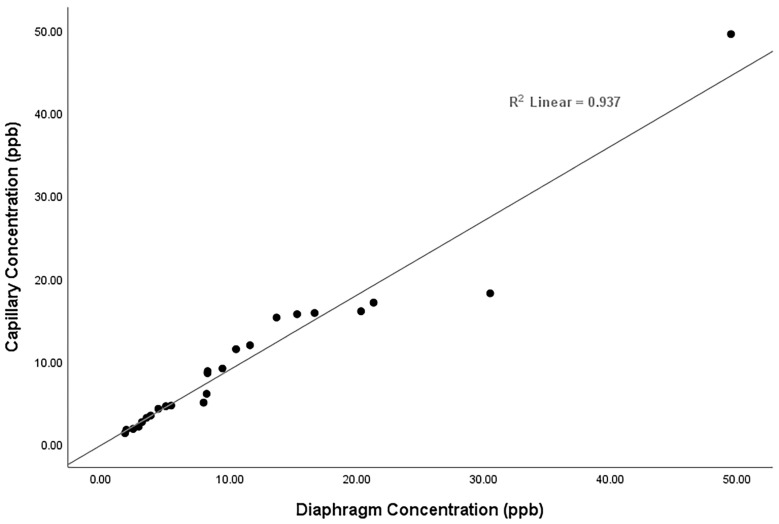
Comparison of all TCE concentrations collected using the diaphragm controller (x-axis) and the capillary controller (y-axis) for all sampling events and all locations (March 2017, May 2017, August 2017, January 2018, May 2018, and August 2018).

Figure 6 shows the regression analysis for the two methods, demonstrating a slope of 0.894 (y = 0.894x + 0.2691) and an R^2^ value of 0.937. The agreement between the two methods was very good and reflects that the time-weighted averages for the 24 h and 14 d samples have a strong correlation for a field-testing comparison. The overall slope does suggest that the capillary, on average, undersampled the 24 h diaphragm samples. Figure 3 and Figure 4, as well as Figures S1–S4, show a similar trend, where 21 of the 24 comparisons demonstrated lower concentrations. 

Co-located 24 h samples were collected for all but the March 2017 sampling event. The co-located samples provide a means of comparison for samplers at the same location. The average difference for the co-located samples was 12.6%, which is a measure of the precision of the diaphragm sampling system (n = 43). The capillary sampling system (14 d) demonstrated similar precision for the paired samplers at each location, with an overall difference of 11.9% (n = 24). Both flow controllers performed well in reliability, with less than a 1% rejection. However, the same research technicians maintained, cleaned, shipped, and analyzed all samples, which may have resulted in a lower failure rate than most field projects. 

An assessment of the interaction of key variables that can influence VOC concentrations from VI as sampled by the two canister methods was conducted using Jmp Statistics 9 JMP (13.2) Software (SAS Institute Inc., Cary, NC, USA). The key factors evaluated included the sampling date, indoor temperature, outdoor relative humidity, interaction of indoor temp and indoor relative humidity, building (location), indoor relative humidity, and location within the building.

The results of the multivariate analysis suggest an association between temperature, relative humidity (RH), and TCE concentration. A ratio for the two types of sampling (14 d and 24 h) was calculated to allow for statistical analysis. If the average is used, then one has n = 1 or 2 for the 14 d samples and n = 14 for the 24 h samples; hence, statistics cannot be performed on the dataset. To resolve the problem with the number of samples, ratios of the 24 h data (diaphragm flow controllers) to the 14 d data (capillary flow controllers) were determined. A strong correlation was observed between the ratio of the diaphragm flow controllers and capillary flow controllers (r = 0.9655). In addition, a reasonable and expected correlation was observed between temperature and humidity (r = 0.6330).

## 4. Discussion

The use of the capillary flow controller for sub-slab sampling over a 24 h period demonstrated the capabilities for sampling over shorter times with smaller canisters (1 L). While the focus of this project was to demonstrate the long-term sampling capabilities using 6 L or larger canisters, the authors recognize the need for shorter sampling periods depending on the scope of the project. Hence, the lower flow rates of the capillary flow controller versus the diaphragm flow controllers allowed for 1, 8, and 24 h samples using lightweight 400 mL or 1 L canisters that can be used for personal and area sampling in VI buildings for shorter time periods [39]. Spatial variation was also observed over 24 h in the sub-slab samples collected each day in Buildings A and B. The variability within Building A was considerable, with sub-slab concentrations in L1 generally between 300 and 800 ppm and L2 between 3 and 22 ppm. In Building B, the concentrations were significantly lower but displayed greater variability between sampling events of up to two orders of magnitude, where sub-slab concentrations for L3 were found to range from 3 to 26 ppm, and L4 was found to have TCE concentrations ranging from less than 0.1 to 15 ppm. The spatial variability was a benefit for this study because it provided evidence that the capillary flow controllers performed well over concentrations that span two orders of magnitude for indoor environments. 

The study design provided an excellent opportunity to collect a robust sample set in different locations and different seasons for both the capillary and diaphragm flow controllers. The study was limited to a single contaminant; however, previous research with canisters and flow controllers has documented their use with multiple chemicals [26,27,28]. 

The use of the capillary system for air sampling, despite advantages such as simplicity and reliability for long-term sampling, has several limitations. The flow rate will start to drop significantly if the canister is filled more than ~50%; thus, it is recommended that the canister be routinely filled to <40% of the canister volume. Uncertainty arises from the system flow rate if the canister is filled beyond 40%, especially if large fluctuations in airborne concentration occur during the latter part of the sampling period. A second limitation is related to the detection limits. If the concentrations of the airborne compounds are low, then it may be difficult to detect all compounds of interest. However, with current analytical equipment, this is rarely a problem for most indoor contaminants. 

Data from this project as well as from other VI investigations suggest that indoor VOC concentrations can fluctuate by two orders of magnitude from one day to another and from season to season. Long-term sampling may be more representative of the long-term average concentration within a building, and thus, relying on one-day averages may result in an under- or overestimation of VI contamination and subsequent exposure. In addition, with shorter sampling times such as 24 h, the timing of the sampling becomes much more critical and can be a limitation in interpreting the results [20]. The ability to collect an air sample over a long period (weeks) of time can enhance VI investigations by providing a larger number of days sampled vs. the traditional 24 h sample. The cost per sample is approximately the same for analysis and shipping, yet the duration of the exposure period observed is significantly greater (1 day vs. 14 days). The results from this study indicate that the capillary-canister can be used to generate a more efficient estimate of exposures for periods greater than 24 h. This expanded sampling period will provide greater flexibility with respect to the timing of the sample and with respect to environmental conditions. 

It is important to note that indoor air sampling is just one step in evaluating buildings impacted by VI; however, it is often a critical parameter for assessing health impacts [41]. Long-term air sampling provides a better estimate of chronic exposure as compared to short-term sampling. It is recognized that long-term air sampling may not always be suitable in circumstances where acute health risks need to be assessed or short-duration indoor source releases occur. The capillary was found to be robust and comparable in cost for a single sample but substantially less expensive than current methods when characterizing a period longer than 24 h. 

## 5. Conclusions

Exposure assessments in indoor environments are complicated by temporal and spatial relationships with multiple sources of contaminants [43], requiring methods that incorporate extended sampling campaigns. The long-term capillary flow controller performed well in the field under significant temporal and spatial variation in indoor air concentrations. The low flow rates (~0.11 and 0.32 mL/min) remained relatively consistent across a broad range of temperature and humidity conditions. The gold standard of comparison for this project was defined based on the concentration observed in the 14 successive 24 h diaphragm samplers. The majority of the tests (22 out of 24) demonstrated that the capillary performed within the 95% confidence interval surrounding the GM of the 24 h samples for each location. While the capillary flow controller performed as well as the diaphragm flow controller with respect to returning similar results, the long-term sampling component provides a capability that was not possible with the 24 h samplers. The ability to collect a sample for a few days up to two weeks provides for an improved characterization (14 days vs. 1 day) for similar resources and effort. In addition, the low flow rate allows for smaller canisters (1 L) to be used for multi-day (3–4 d) sampling. Additionally, the capillary controller performed well when compared to the traditionally used diaphragm flow controller under a broad range of concentration, temperature, and humidity conditions. Developing an accurate VI risk assessment is time-consuming and costly and has often relied upon a suboptimal duration of indoor air sampling as evidence of exposure. Although a VI environment was selected for this study, the methodology, canisters, and low-flow controllers translate to other indoor air and/or industrial air quality settings. An extension of this work could include residential indoor air studies and evaluations in industrial environments. 

## Figures and Tables

**Table 1:** Mean (Standard Deviation) environmental conditions monitored during each sampling event.

Environmental Conditions for Each Event						
	Mar-17	May-17	Aug-17	Jan-18	May-18	Aug-18
Avg. Indoor Temp (°C)	NA	20.5 (0.9)	26.3 (0.8)	6.8 (2.7)	20.1 (1.7)	26.1. (1.4)
Avg Outside Temp (°C)	9.53 (5.8)	21.2 (2.9)	25.4 (2.3)	2.6 (7.5)	20.4 (1.3)	26.6 (1.9)
Temp. Range (°C)	−1.11–18.9	13.3–26.1	21.67–30.1	−11.1–19.4	18.3–22.2	24.4–29.4
Avg. Wind (mph)	8.6 (1.8)	8.2 (3.1)	5.7 (2.2)	7.4 (2.3)	6.6 (1.9)	4.7 (1.6)
Avg Precip. per Day (in.)	0.1 (0.3)	0.2 (0.2)	0.2 (0.4)	0.1 (0.3)	0.3 (0.8)	0.18 (0.4)
Humidity (%)	69.0 (14.6)	78.3 (11.7)	80.5 (4.5)	68.3 (15.0)	64.2 (16.1)	74.9 (9.5)

**Table 2:** Mean and Standard Deviation of indoor concentrations of TCE from 24 h samples at each location for each sampling event.

Dates	Bldg A Midway L1 (ppb)		Bldg A Front Office L2 (ppb)		Bldg B Lunch Room L3 (ppb)		Bldg B Big Room L4 (ppb)	
	AM (SD)	GM (GSD)	AM (SD)	GM (GSD)	AM (SD)	GM (GSD)	AM (SD)	GM (GSD)
Mar 2017	24.9 (18.9)	18.9 (2.19)	9.1 (6.2)	6.9 (2.4)	4.3 (3.7)	3.0 (2.4)	2.3 (1.5)	1.9 (2.0)
May 2017	49.5 (40.7)	38.1 (2.1)	21.3 (18.0)	15.7 (2.3)	13.7 (24.2)	3.6 (4.1)	8.2 (9.7)	3.6 (4.1)
Aug 2017	15.3 (8.5)	13.8 (1.6)	8.2 (5.5)	6.5 (2.1)	4.3 (4.6)	3.1 (2.3)	2.4 (2.3)	1.5 (3.1)
Jan 2018	16.7 (11.5)	14.1 (1.7)	6.7 (5.6)	6.3 (2.3)	10.5 (14.4)	6.9 (2.4)	1.7 (1.4)	1.3 (2.3)
May 2018	11.6 (4.7)	10.7 (1.5)	4.9 (3.2)	3.8 (2.3)	3.1 (2.1)	2.4 (2.2)	1.8 (1.4)	1.3 (2.7)
Aug 2018	20.3 (9.8)	18.8 (1.5)	9.4 (3.4)	8.8 (1.5)	5.4 (4.3)	4.4 (1.8)	3.4 (2.0)	2.8 (2.1)

SD—Standard Deviation; GM—Geometric Mean; GSD—Geometric Standard Deviation.

## Data Availability

The data presented in this study are available on request from the corresponding author. The data are not publicly available due to DoD restrictions.

## Supplementary Materials

The following are available online at https://www.mdpi.com/article/10.3390/ijerph20064811/s1, Sample Collection Plan: Figure S1: Comparative analysis of traditional diaphragm daily samples and capillary controller 14 d samples in four locations (A-L1, B-L2, C-L3, D-L4) during a 2 wk period in March 2017. Gray bars represent daily concentrations, the black dashed line represents the average of the daily samples, black bars represent the 95% confidence interval for the average of the daily samples, and the orange dashed line represents the 14 d sample average. Figure S2: Comparative analysis of traditional diaphragm daily samples and capillary controller 14 d samples in four locations (A-L1, B-L2, C-L3, D-L4) during a 2 wk period in May 2017. Gray bars represent daily concentrations, the black dashed line represents the average of the daily samples, black bars represent the 95% confidence interval for the average of the daily samples, and the orange dashed line represents the 14 d sample average. Figure S3: Comparative analysis of traditional diaphragm daily samples and capillary controller 14 d samples in four locations (A-L1, B-L2, C-L3, D-L4) during a 2 wk period in May 2018. Gray bars represent daily concentrations, the black dashed line represents the average of the daily samples, black bars represent the 95% confidence interval for the average of the daily samples, and the orange dashed line represents the 14 d sample average. Figure S4: Comparative analysis of traditional diaphragm daily samples and capillary controller 14 d samples in four locations (A-L1, B-L2, C-L3, D-L4) during a 2 wk period in August 2018. Gray bars represent daily concentrations, the black dashed line represents the average of the daily samples, black bars represent the 95% confidence interval for the average of the daily samples, and the orange dashed line represents the 14 d sample average.
