# Supplementary material for: Evaluation of Long-Term Flow Controller for Monitoring Gases and Vapors in Buildings Impacted by Vapor Intrusion
Source: Int J Environ Res Public Health. 2023 Mar 9;20(6):4811. doi: 10.3390/ijerph20064811 (PMC10049467; doi:10.3390/ijerph20064811)
Supplement: Supplementary file 1 [file ijerph-20-04811-s001.zip › ijerph-2154659-supplementary.pdf]

Sample Collection Plan

This project demonstrated and validated the use of a commercialized capillary canister sampling system to enable long-term and representative sampling of VOCs in indoor environments impacted by VI. The new canister sampling method provides most of the advantages of both canisters and sorbent samplers and avoids many of their limitations by allowing for long-term (1-3 weeks) sample collection and characterization of VOCs in buildings at risk for VI. Specific aims included:

1. Demonstrate and validate use of evacuated canisters with a capillary flow controller for long-term (~2 weeks) collection of VOCs in a single canister.
2. Compare the performance of the long-term capillary canister sampling system to that of 24 h canister diaphragm sampling methods.
3. Provide data necessary to understand the benefits and limitations of the capillary canister sampling approach.

Performance Objective: Compare Two Week (14 d) to 24 h Canister Sample Concentrations

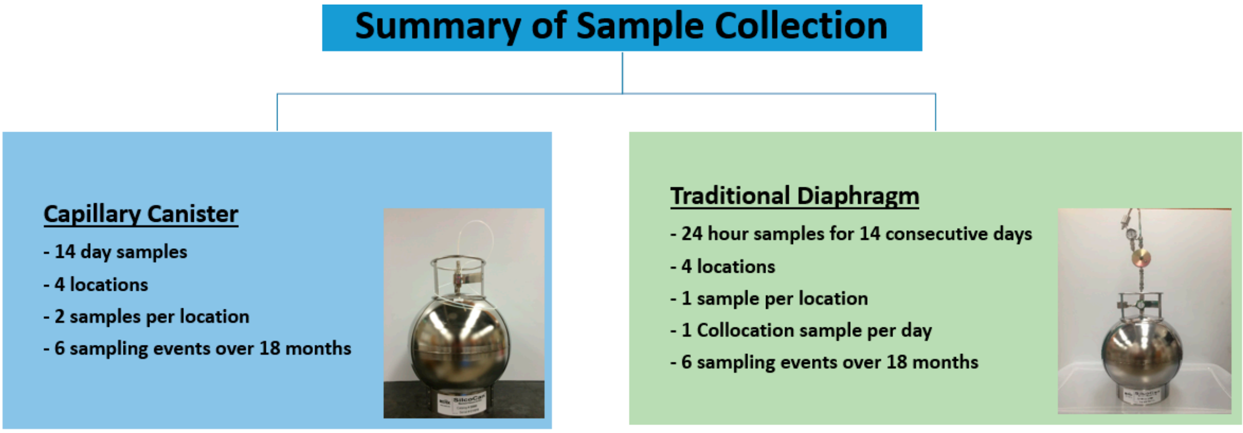

Success Criteria

Substantial reductions in the variance are possible when extending the time of sampling, hence a week long measurement as opposed to daily measurements will provide a better estimate of the actual long term exposure. Capillary canister sampling will be deemed successful relative to 24 h diaphragm sampling if the following criteria are met:

- Temporal relationship between the 14 day canisters and the multiple 24 h canisters for each location will be analyzed using a time-weighted average calculation. Statistical analysis will be performed comparing the two sampling methods.
- The capillary canister approach will not be statistically different with 95% confidence in identification and quantification of indoor air contaminants relative to the 24 h diaphragm canister method.
- Collocated samples will allow for an analysis of how repeatable the 14 day method is with respect to the 24 h canister method. Capillary canister collocated samples will not be statistically different with 95% confidence relative to the 24 h diaphragm canister method.

Suppimental Figures S1-S4 for the additional four sampling events conducted in this study not displayed in the main body of the manuscript: March 2017, May 2017, May 2018 and August 2018

A

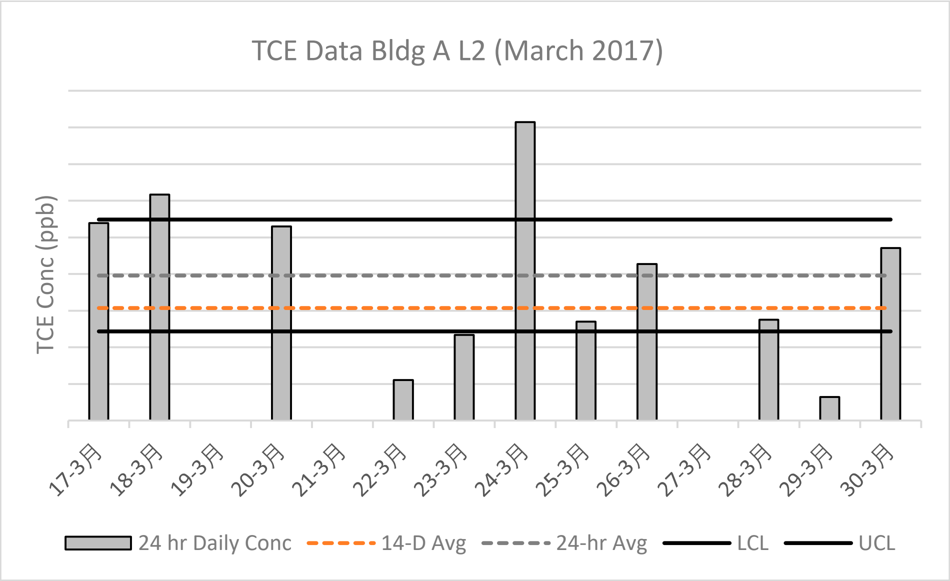

B

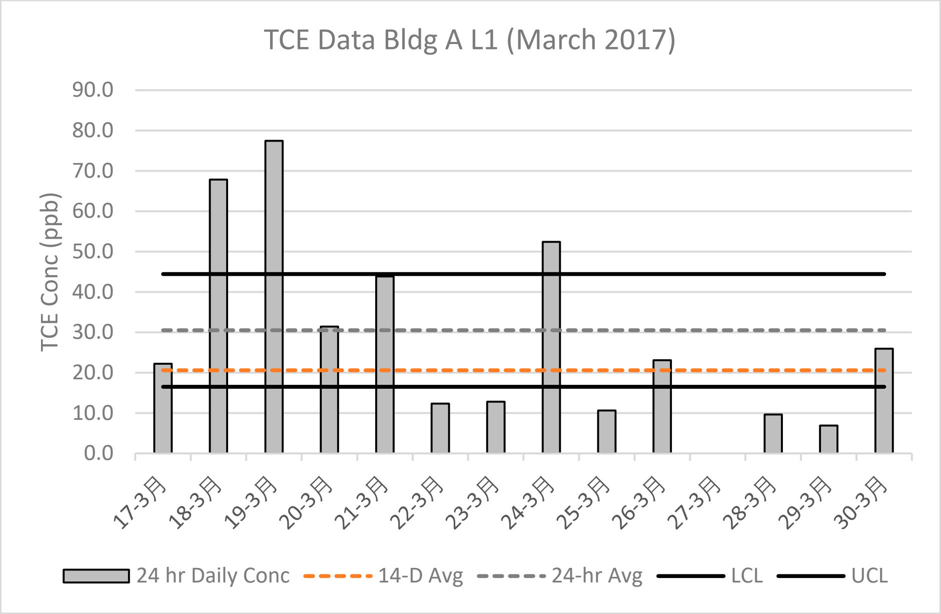

C

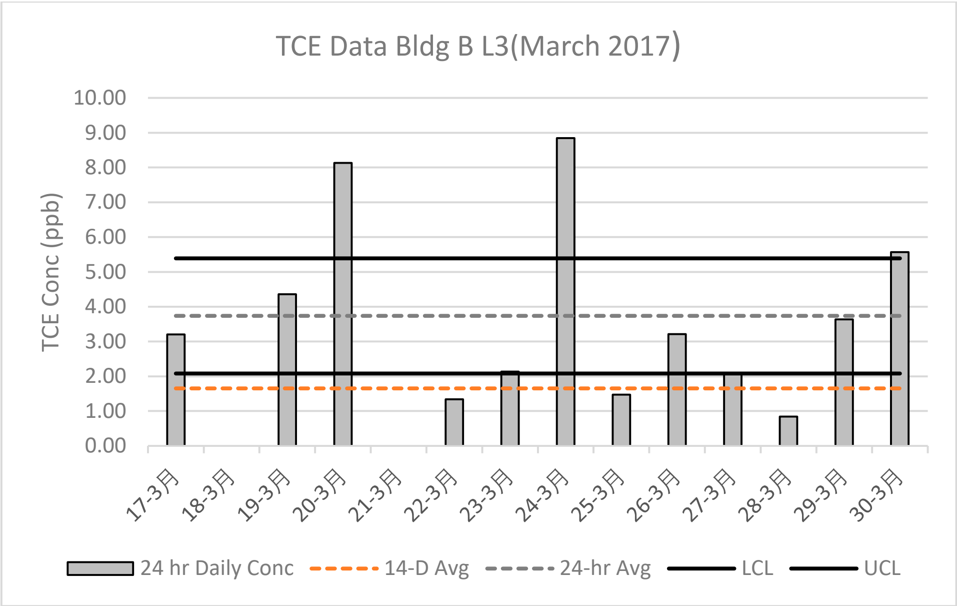

D

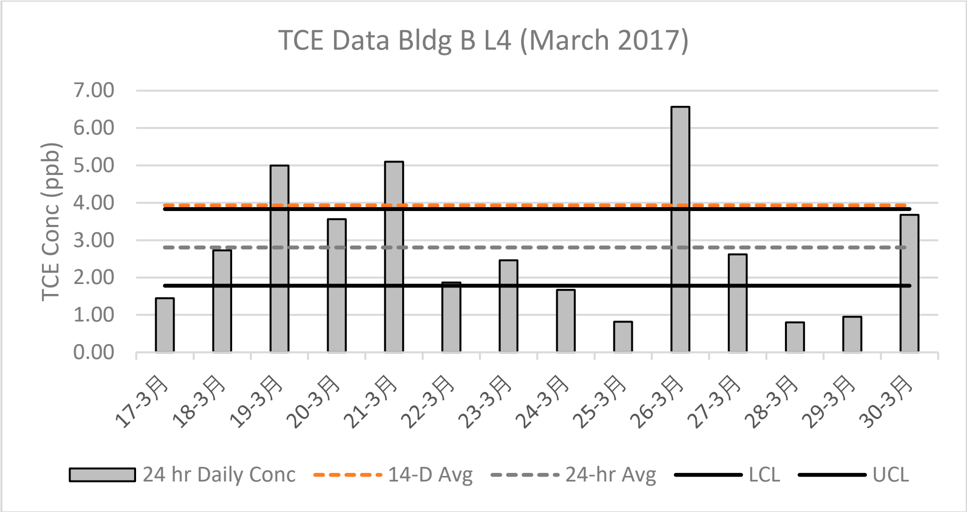

Figure S1. Comparative analysis of traditional diaphragm daily samples and capillary controller 14-d samples in four locations (A-L1, B-L2, C-L3, D-L4) during a 2-week period in March 2017. Grey bars represent daily concentrations, black dashed line represents the average of the daily samples, black bars represent the 95% confidence interval for the average of the daily samples, and the orange dashed line represents the 14-d sample average.

A

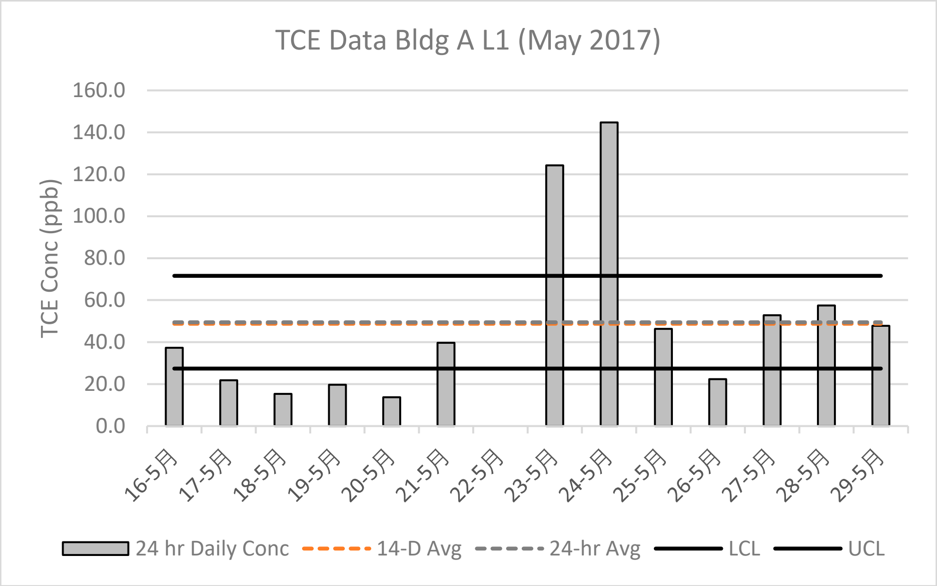
TCE Data Bldg A L1 (May 2017)
TCE Conc (ppb)
160.0
140.0
120.0
100.0
80.0
60.0
40.0
20.0
0.0
16-5月
17-5月
18-5月
19-5月
20-5月
21-5月
22-5月
23-5月
24-5月
25-5月
26-5月
27-5月
28-5月
29-5月
24 hr Daily Conc
14-D Avg
24-hr Avg
LCL
UCL

B

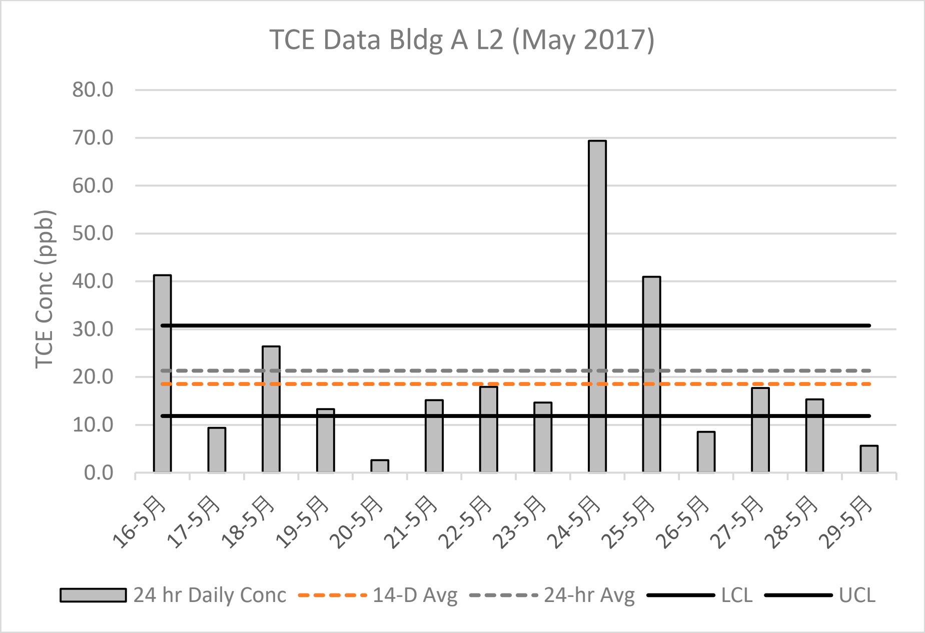
TCE Data Bldg A L2 (May 2017)
TCE Conc (ppb)
80.0
70.0
60.0
50.0
40.0
30.0
20.0
10.0
0.0
16-5月
17-5月
18-5月
19-5月
20-5月
21-5月
22-5月
23-5月
24-5月
25-5月
26-5月
27-5月
28-5月
29-5月
24 hr Daily Conc
14-D Avg
24-hr Avg
LCL
UCL

C

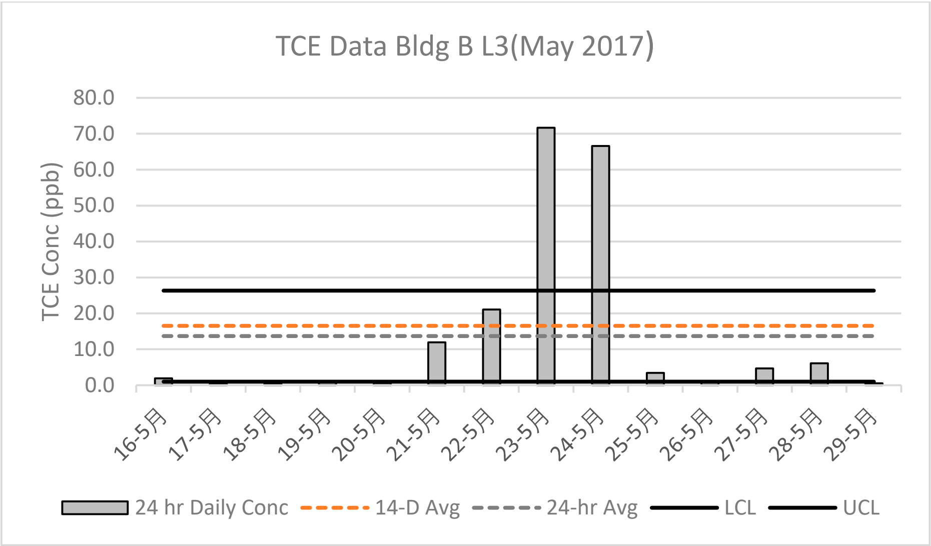

D

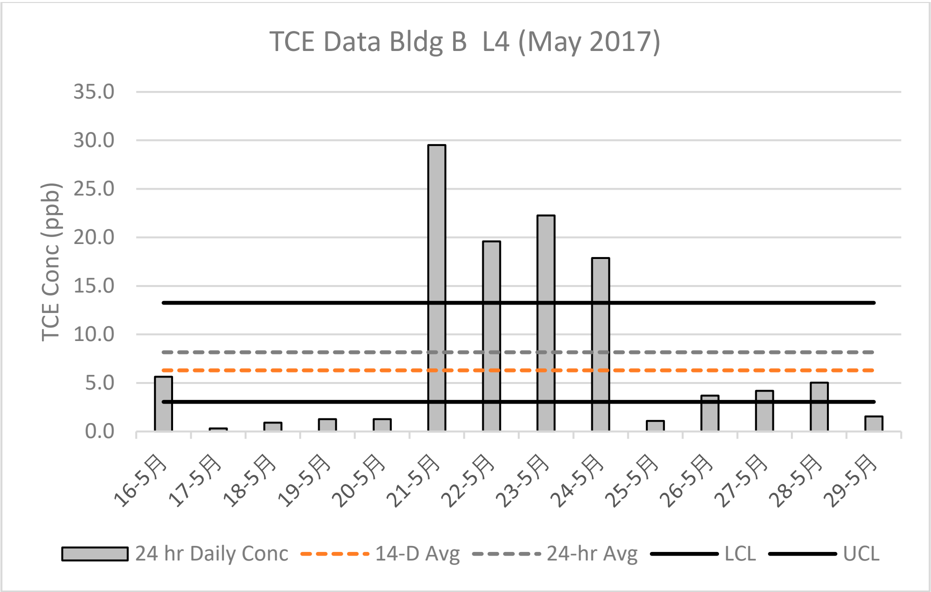

Figure S2. Comparative analysis of traditional diaphragm daily samples and capillary controller 14-d samples in four locations (A-L1, B-L2, C-L3, D-L4) during a 2-week period in May 2017. Grey bars represent daily concentrations, black dashed line represents the average of the daily samples, black bars represent the 95% confidence interval for the average of the daily samples, and the orange dashed line represents the 14-d sample average.

A

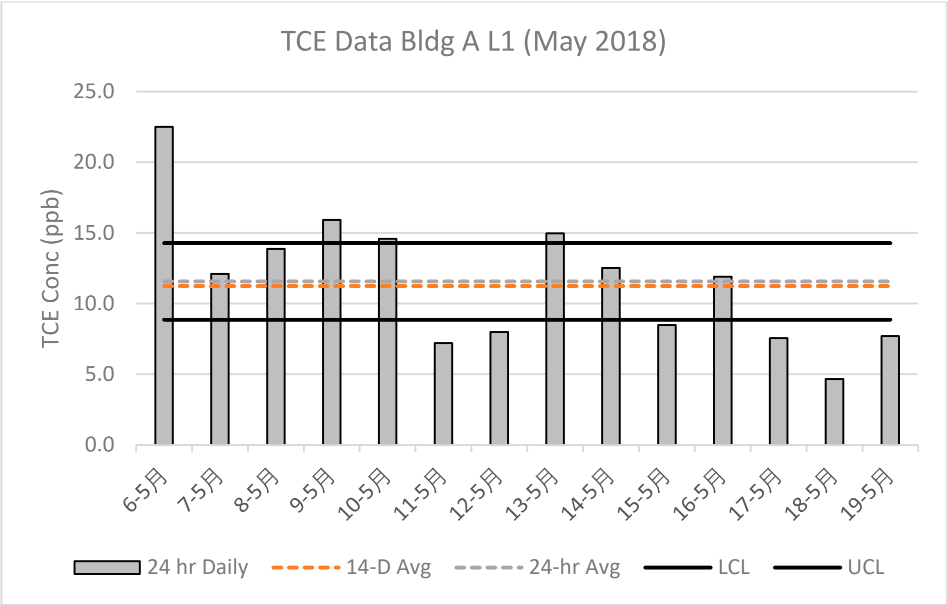
TCE Data Bldg A L1 (May 2018)
TCE Conc (ppb)
25.0
20.0
15.0
10.0
5.0
0.0
6-5月
7-5月
8-5月
9-5月
10-5月
11-5月
12-5月
13-5月
14-5月
15-5月
16-5月
17-5月
18-5月
19-5月
24 hr Daily
14-D Avg
24-hr Avg
LCL
UCL

B

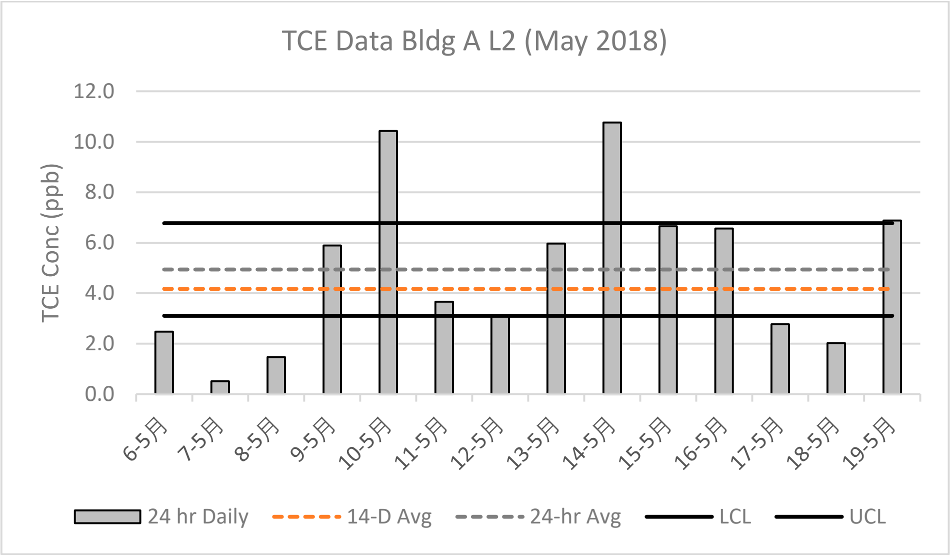
TCE Data Bldg A L2 (May 2018)
TCE Conc (ppb)
12.0
10.0
8.0
6.0
4.0
2.0
0.0
6-5月
7-5月
8-5月
9-5月
10-5月
11-5月
12-5月
13-5月
14-5月
15-5月
16-5月
17-5月
18-5月
19-5月
24 hr Daily
14-D Avg
24-hr Avg
LCL
UCL

C

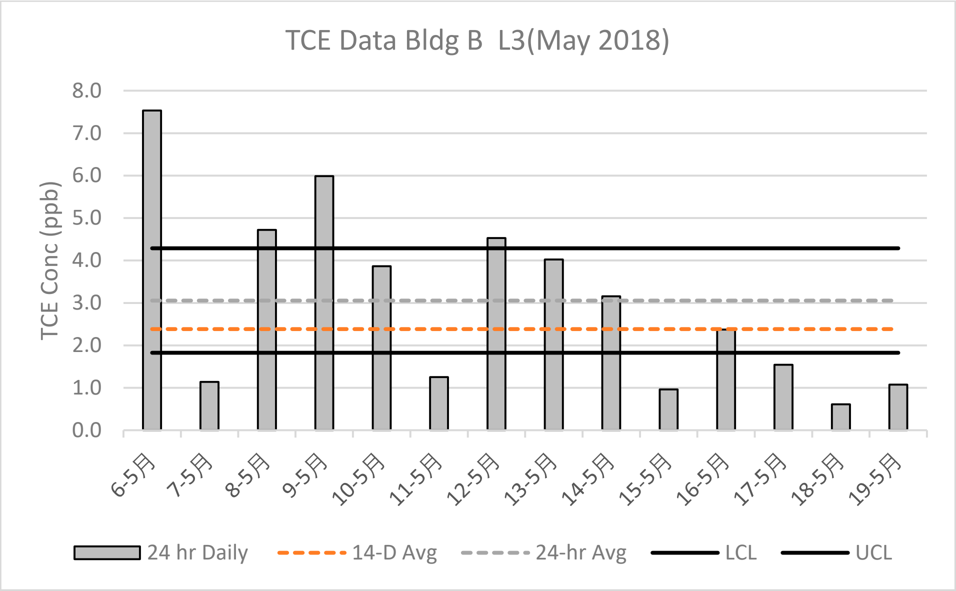

D

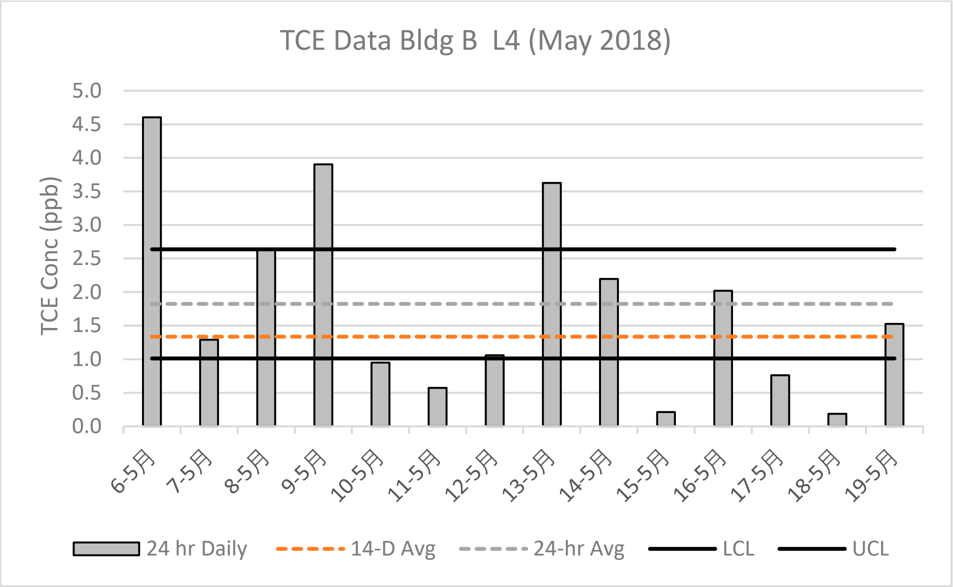

Figure S3. Comparative analysis of traditional diaphragm daily samples and capillary controller 14-d samples in four locations (A-L1, B-L2, C-L3, D-L4) during a 2-week period in May 2018. Grey bars represent daily concentrations, black dashed line represents the average of the daily samples, black bars represent the 95% confidence interval for the average of the daily samples, and the orange dashed line represents the 14-d sample average.

A

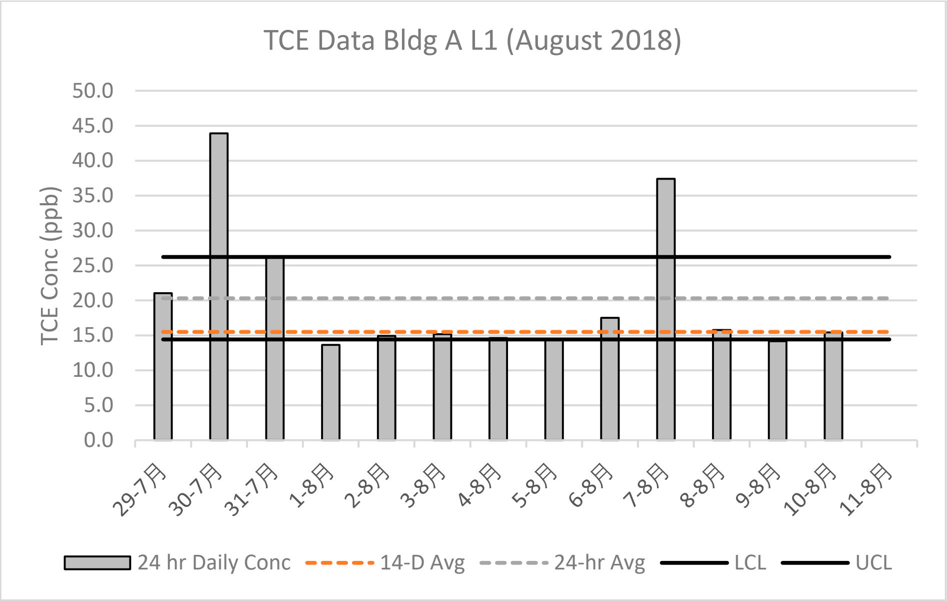
TCE Data Bldg A L1 (August 2018)
TCE Conc (ppb)
50.0
45.0
40.0
35.0
30.0
25.0
20.0
15.0
10.0
5.0
0.0
29-7月
30-7月
31-7月
1-8月
2-8月
3-8月
4-8月
5-8月
6-8月
7-8月
8-8月
9-8月
10-8月
11-8月
24 hr Daily Conc
14-D Avg
24-hr Avg
LCL
UCL

B

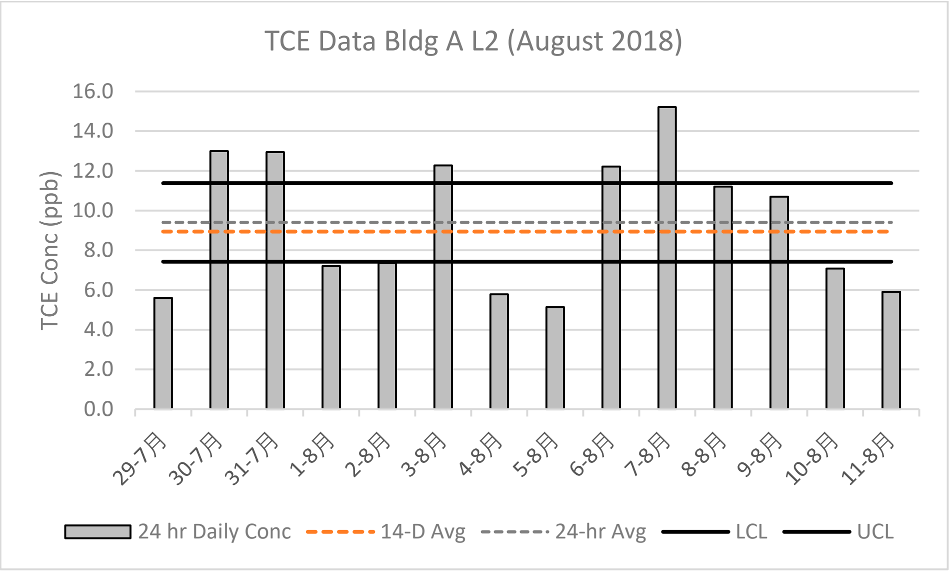
TCE Data Bldg A L2 (August 2018)
TCE Conc (ppb)
16.0
14.0
12.0
10.0
8.0
6.0
4.0
2.0
0.0
29-7月
30-7月
31-7月
1-8月
2-8月
3-8月
4-8月
5-8月
6-8月
7-8月
8-8月
9-8月
10-8月
11-8月
24 hr Daily Conc
14-D Avg
24-hr Avg
LCL
UCL

C

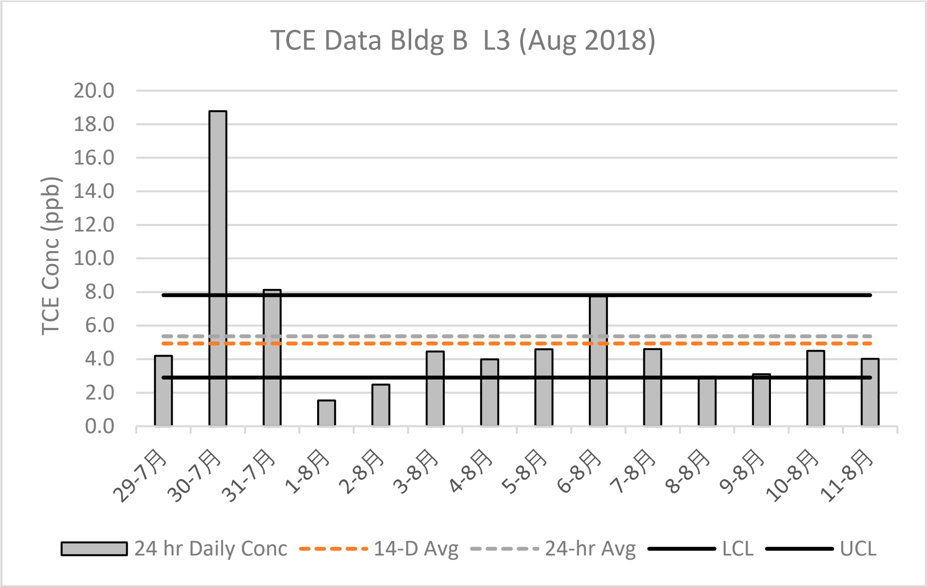

D

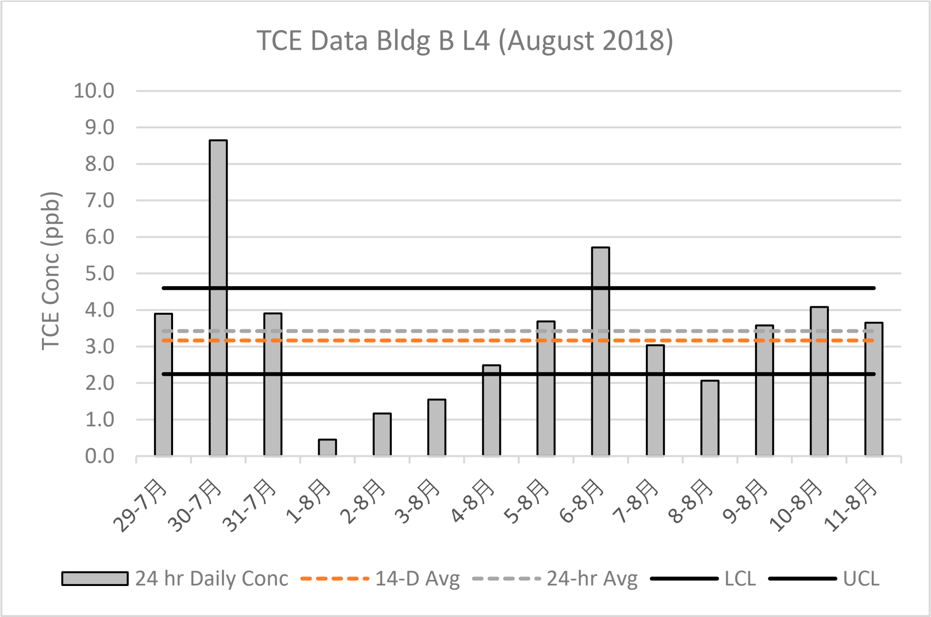

Figure S4. Comparative analysis of traditional diaphragm daily samples and capillary controller 14-d samples in four locations (A-L1, B-L2, C-L3, D-L4) during a 2-week period in August 2018. Grey bars represent daily concentrations, black dashed line represents the average of the daily samples, black bars represent the 95% confidence interval for the average of the daily samples, and the orange dashed line represents the 14-d sample average.
